# Body Composition and Melanoma Outcomes in Patients on Immunotherapy or Targeted Therapy: An Analysis from Canadian Melanoma Research Network

**DOI:** 10.3390/curroncol33070403

**Published:** 2026-07-06

**Authors:** Mohammad Biglari, Sanji Ali, Thiago Muniz, Marcus Butler, Marguerite Ennis, Scott Ernst, Ana Elisa Lohmann

**Affiliations:** 1 Department of Medical Oncology, University of Western Ontario, London, ON N6A 3K7, Canada; mohammad.biglari@lhsc.on.ca (M.B.);; 2 Medical Oncologist, BC Cancer Abbotsford Center, Abbotsford, BC V2S 1J8, Canada; 3 Princess Margaret Cancer Center, Division of Medical Oncology and Hematology, Department of Medicine, University of Toronto, Toronto, ON M5S 1A1, Canada; 4 Applied Statistician, Markham, ON L3R 6H9, Canada; 5 Department of Epidemiology, University of Western Ontario, London, ON N6A 3K7, Canada

**Keywords:** body composition, immunotherapy, melanoma, obesity, targeted therapy

## Abstract

Melanoma remains a major global health burden, though immunotherapy and targeted therapy have markedly improved survival. Obesity has paradoxically been associated with favorable outcomes in melanoma, yet body mass index (BMI) alone fails to capture its influence on treatment response. To address this gap, we conducted a multi-site cohort study within the Canadian Melanoma Research Network, including patients with advanced melanoma treated with immunotherapy or targeted therapy. Body composition was quantified using computerized tomography (CT) imaging to assess visceral adipose tissue (VAT), subcutaneous adipose tissue (SAT), skeletal muscle (SM) mass and intermuscular adipose tissue (IMAT), and associations with progression-free survival (PFS) and overall survival (OS) were evaluated. No overall association was seen for BMI, SAT, VAT, IMAT or SM with PFS or OS. In the targeted therapy subset, higher BMI, SAT, VAT and SM were associated with better OS (hazard ratios 0.56 to 0.65), while no effect was seen in the immunotherapy group. IMAT emerged as a novel prognostic marker, with elevated levels associated with lower OS in males and better OS in females. Our findings show that CT-based body composition is not associated with survival outcomes in patients with advanced melanoma receiving immunotherapy.

## Simple Summary

Melanoma is a serious skin cancer that continues to cause illness and death worldwide, but modern treatments such as immunotherapy and targeted therapy have greatly improved survival. Obesity has long been associated with cancer risk and immune dysfunction, yet in melanoma it seems to improve outcomes—a phenomenon known as the “obesity paradox.” Body mass index (BMI) cannot distinguish between fat types or muscle mass, which may be critical for understanding treatment response. In this large Canadian study, we used computerized tomography (CT) scans to measure body composition more precisely. We found that the obesity paradox was restricted to patients receiving targeted therapy, and this was mirrored in the body composition measurements. Our results show that CT-based body composition is not associated with survival outcomes in patients with advanced melanoma receiving immunotherapy.

## 1. Introduction

Worldwide, melanoma affects many people; around 325,000 individuals were diagnosed in 2020, with 57,000 losing their lives [1]. About 11,300 new diagnoses had been estimated by 2024 in Canada, along with 1300 related mortalities [2]. In patients with unresectable or advanced melanoma, systemic therapies—particularly immunotherapy and targeted therapy—have significantly improved progression-free survival (PFS) and overall survival (OS) [3,4,5,6]. Mutations in the RAS-RAF-MAPK pathway are commonly observed in melanoma, with BRAF mutations present in almost 60% of advanced cases, making targeted therapy effective in these cases [7,8,9,10,11]. Immunotherapy, however, is effective for patients regardless of BRAF status [4,6].

Obesity, conventionally measured by body mass index (BMI), is a known risk factor for several malignancies, including melanoma [12]. When it comes to treatment outcomes in skin cancer such as melanoma, its role remains controversial, with studies reporting both detrimental and paradoxically favorable outcomes [12,13,14]. Immune aging, PD-1-mediated T-cell dysfunction, and tumor-promoting metabolic effects are present in obesity [15]. Nevertheless, retrospective analyses have reported improved outcomes in obese patients treated with immunotherapy (HR 0.75 [0.56–1.00] for PFS; HR 0.64 [0.47–0.86] for OS) and targeted therapy (HR 0.72 [0.57–0.91] for PFS; HR 0.60 [0.45–0.79] for OS) [16]. These findings suggest a paradoxical obesity effect, though BMI does not accurately reflect fat distribution and lean mass, which may significantly affect treatment response [17].

Body composition analysis provides a more precise understanding of obesity-related mechanisms beyond what BMI can indicate. Visceral adipose tissue (VAT) is metabolically active and may interfere with cancer biology through insulin resistance, proinflammatory cytokines, angiogenesis and immune dysregulation [18,19,20]. In contrast, subcutaneous adipose tissue (SAT) secretes higher levels of leptin and estradiol, modifying immune function and tumor behavior [21,22,23]. BMI cannot differentiate between VAT, SAT or skeletal muscle mass [24]. Despite emerging interest, the relationship between immune checkpoint inhibitor (ICI) outcomes and specific body composition parameters such as muscle mass and adipose tissue distribution is not clearly defined in patients with metastatic melanoma.

This study evaluates the association between body composition—shown through computed tomography (CT) imaging—BMI and clinical outcomes in patients with unresectable or metastatic melanoma treated with immunotherapy or targeted therapy, using data from the Canadian Melanoma Research Network. Considering central adiposity and muscle mass, we aim to investigate the physiological mechanisms underlying the obesity paradox and hypothesize that higher subcutaneous fat is associated with improved PFS and OS.

## 2. Materials and Methods

This population-based cohort study examined data from patients with unresectable or metastatic melanoma at the Verspeeten Family Cancer Centre, London, Ontario and the Princess Margaret Cancer Centre, Toronto, Ontario, Canada, from March 2010 to August 2021. We used data from the Canadian Melanoma Research Network—a national registry that collects prospective data from 13 cancer centers across Canada.

Patients aged 18 years of age or older were eligible if they had histologically confirmed unresectable or metastatic melanoma and had been treated with either immunotherapy or targeted therapy. They had to have an abdominopelvic CT scan available for assessment of body composition done within 6 months of treatment initiation. The original protocol and all later amendments were approved by the Western University Health Science Research Ethics Board.

The extracted variables included age, gender, height, weight, lactate dehydrogenase (LDH) level, stage at diagnosis, BRAF status, metastatic site, Eastern Cooperative Oncology Group (ECOG) performance status, type of treatment and cause of death. The dates of diagnosis, treatment initiation and progression or death were also recorded. BMI was calculated using height and weight measured by clinic staff at the time of treatment initiation.

We used CT images to manually locate third lumbar vertebra and analyze body composition using muscle and adipose-tissue cross-sections. A validated software program developed at the University of Waterloo, called Automated Muscle and Adipose Tissue Composition Analysis (AutoMATICA), was used for automated segmentation and to quantify body composition, including skeletal muscle (SM) area, intermuscular adipose tissue (IMAT), SAT and VAT, expressed as surface area in squared centimeters [25]. The skeletal muscle index (SMI) was calculated as skeletal muscle area/height2.

AutoMATICA is a deep-learning-based software program that provides automated tissue segmentation at the level of the third lumbar vertebra (L3), a validated anatomical landmark for estimating whole-body composition. The single axial L3 slice was selected according to standard radiologic criteria, and both contrast-enhanced and non-contrast CT scans were accepted; prior validation studies have shown negligible differences in muscle and adipose quantification across contrast phases [26,27]. Segmentation was fully automated, but each case was visually reviewed by trained analysts to ensure accuracy. Quality control included exclusion of scans with artifacts, inadequate field of view, or poor image quality. AutoMATICA has been externally validated in oncology cohorts, demonstrating high concordance with manual segmentation and reproducibility across institutions [25,28].

The primary endpoint of this study was progression-free survival (PFS) which was defined as time from treatment initiation to progression or death in years. If the patient had not progressed, the time is censored at the date patient was last known to be progression-free. The secondary endpoint was overall survival measured in years from treatment initiation until death. If the patient was still alive, the time was censored at the date last known to be alive.

Categorical variables were presented as frequencies and percentages while medians and inter-quartile ranges (IQR) were used for continuous variables. Correlations were calculated using the Spearman rank method.

Cox proportional hazard models were used to analyze the associations of BMI, VAT, SAT, IMAT, SM and SMI with the survival outcomes of PFS and OS. To reduce skewness, BMI, SM and SMI were log-transformed and VAT, SAT and IMAT square-root-transformed before being entered into the models as continuous variables. Hazard ratios (HRs) and 95% confidence intervals (CIs) were reported, where the hazard ratios were calculated for the 75th versus 25th percentiles of the distribution of each body composition variable. These can be found as the end points of the interquartile ranges presented in Table 1.

Main-effects models were fitted for each variable in turn. We then assessed whether the associations differed by treatment type (targeted vs. immunotherapy) and sex (male vs. female) by including body composition-by-treatment and body composition-by-sex interaction terms. In no case were both interactions significant at the same time, so we report here the results of separate interaction models: first, allowing for treatment differences when male and female patients are combined, and then allowing for sex differences when treatment groups are combined.

All models were adjusted for age, sex, treatment type, ECOG performance status (<2 or ≥2) and LDH level. Since cancer stage may be related to both body composition and survival, we did not include it in our primary models. However, we included stage (grouped as III/M1a/M1b vs. M1c vs. M1d) as an additional covariate in the Supplementary Materials.

All statistical analyses were performed using R software (version 4.5.1; R Core Team (2025); R: A language and environment for statistical computing. The statistical significance level was defined as a *p*-value less than 0.05.

## 3. Results

**Figure 1:**
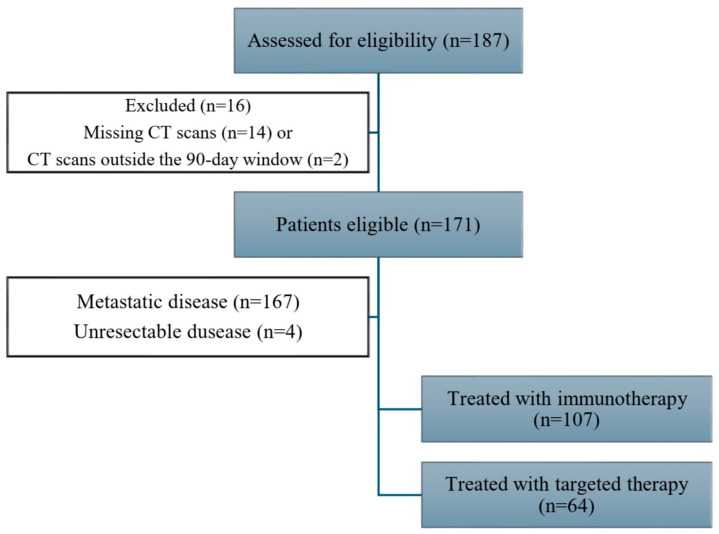
Patient flow chart.

In total, we identified 187 patients with unresectable or metastatic melanoma treated with immunotherapy or targeted therapy for review (Figure 1). Fourteen patients were missing CT data, and two patients had their CT scan done outside the 6-month window specified in the eligibility criteria. Hence, 171 patients were included in this study from the two cancer centers. CT scans were performed a median of 21 days before treatment initiation, with an interquartile range of to 41.5 to 9 days before treatment initiation.

Patients’ demographic data and tumor characteristics are presented in Table 1. The median age of the study population at treatment initiation was 62.2 years (range 26 to 92). Overall, males made up 64.9% of the study population (N = 111). A total of 62.6% of patients received immunotherapy as a checkpoint inhibitor monotherapy (nivolumab, pembrolizumab, ipilimumab) or a combination of anti-PD-1 antibody with CTLA-4 inhibitor (ipilimumab plus nivolumab or pembrolizumab). Targeted therapy regimens were either a BRAF inhibitor (vemurafenib) or a combination of a BRAF inhibitor/MEK inhibitor (vemurafenib/cobimetinib or dabrafenib/trametinib). Nearly 85% of patients in each group had their treatment first-line therapy. Most patients (97.7%) had metastatic melanoma.

The median BMI of the study population was 27.2 kg/m^2^ (range 14.7 to 57.4), with 28.1% obese (defined as BMI ≥ 30 kg/m^2^). BMI showed a very clear correlation with VAT and SAT (r = 0.8, r = 0.74, respectively) and somewhat lower correlations with SM and SMI (r = 0.43 for both); all correlations were statistically significant (*p* < 0.0001). LDH levels, known to be among the strongest predictors of recurrence, did not show any meaningful correlation with body composition measures and BMI (all |r| ≤ 0.12).

The median follow-up in this study was 5.7 years (95% CI 5.4–6.6). Overall, 148 (86.5%) patients experience disease progression, and 126 (73.7%) died. In the adjusted models, the targeted therapy group had worse PFS and OS than the immunotherapy group (HR = 1.59, 95% CI [1.09–2.32], *p* = 0.015 and HR = 1.39, 95% CI [0.93–2.08], *p* = 0.10), whereas no significant differences were observed for male and female patients (HR = 1.06, 95% CI [0.73–1.54], *p* = 0.75 for PFS and HR = 0.89, 95% CI [0.60–1.32], *p* = 0.57 for OS).

The associations of BMI and body composition with progression or death in the full data set are given in Table 2. Testing our main hypothesis, SAT was not associated with PFS (HR = 1.03, 95% CI [0.82–1.28]; *p* = 0.83) or OS (HR = 0.93, 95% CI [0.74–1.17]; *p* = 0.54). Similarly, no overall association was seen for BMI, VAT, IMAT, SM and SMI with PFS or OS.

Interaction models were used to explore if the association of BMI and body composition with PFS and OS differed by treatment (Table 3), for male and female patients combined. For OS, significant or near-significant interactions (*p* = 0.01 to 0.064) were observed for BMI and all body composition measures except IMAT, such that in the targeted therapy group, higher BMI, SAT, VAT, SM and SMI were associated with better OS (HRs 0.56 to 0.65), whereas no effect was seen in the immunotherapy group (all HRs near 1). SM and SMI showed a similar pattern for PFS.

**Figure 2:**
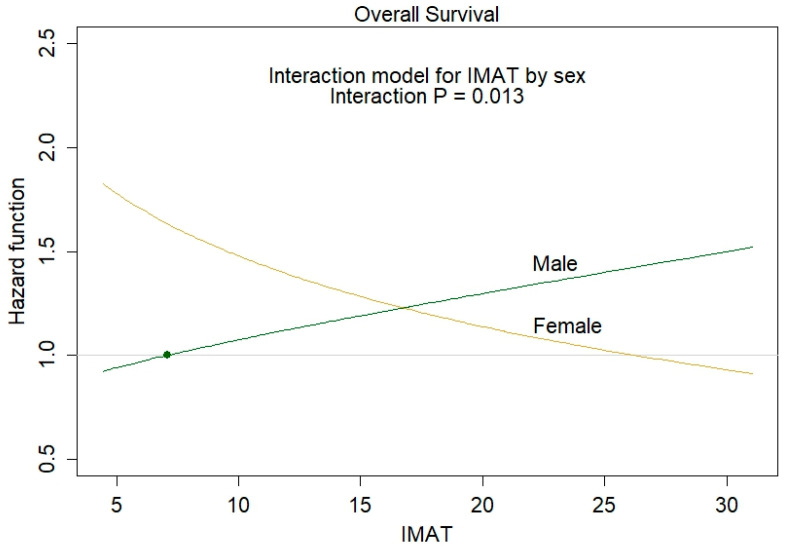
Hazard functions for death in male and female patients as a function of IMAT, derived from an interaction model and combining both treatments. The hazard functions are expressed relative to the hazard for a male patient with IMAT at its 25th percentile (dotted point).

Table 4 provides the model results when associations were allowed to vary by sex for both treatment groups combined. A significant interaction (*p* = 0.013) was seen for IMAT in OS, where women with higher IMAT had better OS than women with lower IMAT (HR = 0.70, 95% CI [0.46–1.05]), whereas the opposite was seen for men (HR = 1.30, 95% CI [0.94–1.79]) (Figure 2).

In supplementary analyses where we adjusted for cancer stage in addition to age, sex, treatment type, ECOG status, and LDH level, the lack of association between BMI/body composition and survival in the overall cohort remained unchanged. Adjustment for stage attenuated the treatment–body composition interaction, resulting in weaker interaction *p*-values, whereas the sex–IMAT interaction became stronger. Full model estimates are provided in Supplementary Tables S1–S3 and Figure S1.

## 4. Discussion

In this cohort of patients with advanced melanoma treated with immunotherapy or targeted therapy, we found that obesity-related measures—including BMI, adipose tissue compartments and skeletal muscle index—were not associated with progression-free survival and overall survival in the overall population. However, in the subset receiving targeted therapy, higher adiposity and greater skeletal muscle mass were associated with improved overall survival. Additionally, higher intermuscular adiposity (IMAT) was associated with better survival in women but tended toward worse survival in men. These findings highlight that the prognostic relevance of body composition in advanced melanoma may be context-dependent, varying by treatment modality and patient sex.

The absence of a survival association with BMI or body composition in the full cohort contrasts with some earlier reports describing an “obesity paradox” in melanoma, particularly in patients receiving immune checkpoint inhibitors (ICIs) [16,29]. However, these findings have been inconsistent, and many analyses rely solely on BMI, which cannot distinguish between adipose compartments or account for muscle mass. Our results add to the growing evidence that BMI alone is an inadequate prognostic marker in melanoma and that the obesity paradox may not be generalizable across all treatment settings.

In the targeted therapy subgroup, higher adiposity and greater skeletal muscle mass were associated with improved overall survival. These findings align with prior reports showing that overweight and obese patients receiving BRAF/MEK inhibitors have longer survival than normal-weight individuals [16,30,31]. Several mechanisms may explain this pattern. Targeted therapies have distinct pharmacokinetic and pharmacodynamic properties, and greater adiposity or muscle mass may reflect enhanced physiologic reserve, improved drug distribution or better tolerance of treatment-related toxicities [32]. Moreover, adipose tissue is metabolically active and influences systemic inflammation, insulin signaling, and cytokine production, which may interact with MAPK pathway activity [33]. Although our study was not designed to evaluate mechanisms directly, the consistency of the association across multiple fat tissues and muscle measures suggests that body composition may impact targeted therapy outcomes through integrated metabolic and physiologic pathways.

In contrast, we did not observe survival benefit associated with higher adiposity or muscle mass in patients receiving immunotherapy. This divergence between treatment modalities may help to reconcile conflicting findings in the literature. Obesity is associated with chronic inflammation, altered T-cell function and increased expression of immune checkpoints such as PD-1, TIM-3 and LAG-3 [34,35,36]. These immunologic alterations may counteract or obscure any potential benefit of increased adiposity in the ICI setting. Some studies have reported improved outcomes among obese patients receiving ICIs [16], while others have found no association [37,38]. Our results suggest that the prognostic impact of obesity and body composition may be attenuated or absent in the immunotherapy context, emphasizing the importance of evaluating body composition within specific therapeutic frameworks rather than assuming uniform effects across treatment classes.

Another finding was the sex-specific association between IMAT and survival. We observed a significant interaction in which higher IMAT was associated with improved survival in women but tended toward worse survival in men. This pattern is in line with prior works suggesting that the obesity paradox may be more pronounced in males [16,31]. Sex differences in immune function, adipose biology, and muscle quality are well documented. Men and women differ in fat distribution, inflammatory profiles and T-cell responses, and these differences may modify the prognostic relevance of muscle quality and adipose infiltration [39,40,41]. Although the mechanisms underlying the sex-specific IMAT association remain unclear, IMAT may reflect distinct metabolic or immunologic states in men versus women. Given that IMAT is associated with insulin resistance, chronic inflammation, and impaired muscle regeneration in other disease settings [42,43,44,45,46,47], its sex-dependent prognostic implications in melanoma warrant further investigation.

When we explored the potential impact of cancer stage by further adjusting our models for stage III, M1a–M1d categories, the overall null association between body composition and survival remained, but some interaction estimates changed in magnitude. Given the strong correlation between stage, LDH, and disease burden, these shifts may reflect overadjustment or collinearity rather than true modification of the underlying associations. Accordingly, we present these M-stage–adjusted models as supplementary, hypothesis-generating analyses.

IMAT itself represents a novel and understudied biomarker in melanoma. Unlike visceral or subcutaneous fat, IMAT reflects ectopic fat deposition within muscle compartments and may capture aspects of muscle quality, metabolic dysfunction, and systemic inflammation not reflected in traditional body composition measures. Our finding that higher IMAT was associated with improved survival in women is particularly intriguing because IMAT is typically associated with adverse metabolic and cardiovascular outcomes—including type 2 diabetes, metabolic syndrome, coronary microvascular dysfunction, and major adverse cardiovascular events—independent of overall adiposity [43,44,45,46,47]. This counterintuitive pattern suggests that IMAT may carry different biological significance in the context of melanoma, potentially reflecting treatment tolerance, preserved physiologic reserve, or sex-specific immune–metabolic interactions. While the mechanism is still unclear, prior studies have shown that IMAT is associated with elevated IL-6 and TNF-α levels, cytokines known to influence melanoma progression and treatment response [43]. The identification of IMAT as a prognostic marker in advanced melanoma is therefore a meaningful contribution. Nevertheless, these IMAT-related associations should be interpreted cautiously, as they remain exploratory and require confirmation in larger, prospective studies.

Taken together, our findings emphasize the limitations of BMI as a prognostic tool in advanced melanoma. CT-based body composition analysis offers a more precise and clinically relevant assessment of adiposity and muscle characteristics and may improve risk stratification and treatment planning. Incorporating such measures into clinical practice could help identify patients who may benefit from supportive interventions aimed at optimizing muscle mass or metabolic health.

Although CT-based body composition assessment is a major strength of this study, several methodological considerations warrant attention. First, although the median interval between CT imaging and treatment initiation was relatively short, the allowable window of up to 6 months within treatment initiation introduces heterogeneity in the timing of body composition measurement. This raises the possibility of selection or survivorship bias. Second, because only a single pre-treatment CT scan was analyzed, our study could not capture dynamic changes in muscle or adipose tissue that may occur due to cancer-related cachexia or weight loss. These temporal and biological factors may impact the associations between body composition and clinical outcomes.

This study has several strengths, including its large, multi-site Canadian cohort, the use of BMI as a continuous variable to enhance statistical precision and avoid arbitrary categorization, and the inclusion of treatment- and sex-specific analyses. Assessment of IMAT as a body composition measure represents a novel contribution to the melanoma literature. Moreover, the cohort’s average BMI was in the normal range, enabling us to evaluate the role of body composition beyond overt obesity. Limitations include the relatively small subgroup with targeted therapy, the retrospective design, and the absence of uniform inflammatory or metabolic biomarkers. Despite these limitations, the consistency of the findings across multiple body composition metrics and the biologically plausible treatment- and sex-specific patterns support the robustness of the results.

## 5. Conclusions

In our multi-site Canadian cohort of patients with advanced melanoma, we found that the prognostic significance of obesity-related measures depends on treatment modality and patient sex. The observed sex-specific difference further highlights the biological complexity of obesity in melanoma. Our novel findings on IMAT suggest that muscle quality may also influence therapeutic outcomes. These insights challenge conventional assumptions and support a multidimensional approach to body composition profiling, leading to more personalized and biologically informed melanoma care.

## Figures and Tables

**Table 1:** Patient and tumor characteristics, N = 171.

Patient Characteristics	
**Age at treatment initiation (years)**	N (%)
<60	73 (42.7%)
≥60	98 (57.3%)
**Sex**	
Male	111 (64.9%)
Female	60 (35.1%)
**AJCC stage**	
III	5 (2.9%)
IV	166 (97.1%)
**Metastatic Stage IV**	
M1a	21 (12.6%)
M1b	33 (19.9%)
M1c	68 (41.0%)
M1d	44 (26.5%)
**ECOG performance status**	
0–1	153 (89.5%)
2–4	18 (10.5%)
**Treatment type**	
Immunotherapy	107 (62.6%)
Targeted therapy	64 (37.4%)
**Immunotherapy type**	
Anti-PD-1	66 (62%)
CTLA-4 inhibitor	1 (1%)
Anti-PD-1 plus CTLA-4 inhibitor	40 (37%)
**Immunotherapy Line**	
1st Line	91 (85%)
≥2nd Line	16 (15%)
**Targeted therapy type**	
BRAF inhibitor	27 (42%)
BRAF inhibitor/MEK inhibitor	37 (58%)
**Targeted therapy line**	
1st Line	54 (84%)
≥2nd Line	10 (16%)
**Body composition (surface area, cm^2^)**	median (IQR)
VAT	139.8 (58.4, 238.2)
SAT	182.3 (131.4, 264.2)
IMAT	(13.3 (7.1, 20.1)
SM	141.5 (116.1, 167.3)
SMI	47.5 (41.2, 56.2)
BMI (kg/m^2^)	27.2 (23.6, 31.2)
**Tumor Characteristics**	
**BRAF status**	N (%)
Negative	59 (34.5%)
Positive	107 (62.6%)
Unknown	5 (2.9%)
**LDH (IU/L)**	
≤225	75 (46%)
226–450	54 (33.1%)
>450	34 (20.9%)
**Liver metastasis**	
Yes	71 (41.5%)
No	100 (58.5%)
**Brain metastasis**	
Yes	42 (24.6%)
No	129 (75.4%)

AJCC: American Joint Committee on Cancer; BMI: body mass index; ECOG: Eastern Cooperative Oncology Group; IMAT: intermuscular adipose tissue; IQR: inter-quartile range; SAT: subcutaneous adipose tissue; SM: skeletal muscle; SMI: skeletal muscle index; VAT: visceral adipose tissue.

**Table 2:** Overall hazard ratios for progression and death from adjusted survival models.

	Progression		Death	
Variable	HR * (95% CI)	*p* Value	HR * (95% CI)	*p* Value
BMI	1.00 (0.78–1.3)	0.982	0.86 (0.67–1.12)	0.261
SAT	1.03 (0.82–1.28)	0.828	0.93 (0.74–1.17)	0.540
VAT	0.97 (0.73–1.29)	0.834	0.87 (0.66–1.17)	0.360
SM	1.08 (0.74–1.57)	0.697	0.91 (0.61–1.35)	0.647
SMI	1.11 (0.8–1.55)	0.522	0.93 (0.65–1.33)	0.686
IMAT	1.02 (0.81–1.3)	0.858	1.01 (0.78–1.3)	0.966

Models adjusted for age, sex, treatment, ECOG status and LDH level. * HRs are for the 75th vs. the 25th percentiles of the distribution of each body composition measure. BMI: body mass index; CI: confidence interval; ECOG: Eastern Cooperative Oncology Group; HR: hazard ratio; IMAT: intermuscular adipose tissue; LDH: lactate dehydrogenase; SAT: subcutaneous adipose tissue; SM: skeletal muscle; SMI: skeletal muscle index; VAT: visceral adipose tissue.

**Table 3:** Body composition and BMI association with patients’ outcomes by treatment type, for male and female patients combined.

Progression-Free Survival							
	Immunotherapy			Targeted Therapy			
	HR *	95% CI	*p*	HR *	95% CI	*p*	Interaction *p*
BMI	1.14	0.82–1.58	0.431	0.84	0.57–1.24	0.383	0.228
SAT	1.11	0.85–1.45	0.458	0.91	0.65–1.27	0.563	0.340
VAT	0.95	0.65–1.38	0.777	1	0.67–1.49	0.992	0.849
SM	1.33	0.87–2.04	0.186	0.79	0.49–1.27	0.336	0.038
SMI	1.45	0.98–2.14	0.0635	0.73	0.46–1.18	0.202	0.016
IMAT	0.98	0.73–1.32	0.894	1.09	0.77–1.52	0.636	0.632
**Overall Survival**							
	**Immunotherapy**			**Targeted Therapy**			
	HR *	**95% CI**	***p***	HR *	**95% CI**	***p***	**Interaction *p***
BMI	1.06	0.76–1.47	0.727	0.65	0.44–0.96	0.029	0.055
SAT	1.07	0.82–1.4	0.61	0.71	0.49–1.02	0.0633	0.064
VAT	1.17	0.8–1.7	0.418	0.61	0.4–0.93	0.0211	0.020
SM	1.15	0.75–1.76	0.52	0.56	0.33–0.96	0.0344	0.010
SMI	1.13	0.75–1.68	0.563	0.61	0.35–1.06	0.0797	0.050
IMAT	1.07	0.79–1.47	0.65	0.91	0.62–1.33	0.619	0.472

Models adjusted for age, sex, treatment, ECOG status and LDH level. * HRs are for the 75th vs. the 25th percentile of each body composition measure. BMI: body mass index; CI: confidence interval; ECOG: Eastern Cooperative Oncology Group; HR: hazard ratio; IMAT: intermuscular adipose tissue; LDH: lactate dehydrogenase; SAT: subcutaneous adipose tissue; SM: skeletal muscle; SMI: skeletal muscle index; VAT: visceral adipose tissue.

**Table 4:** Association of body composition and BMI with patients outcomes by sex, for both treatment groups combined.

Progression-Free Survival					
	Female		Male		
	HR * 95% CI	*p*	HR * 95% CI	*p*	Interaction *p*
BMI	0.93 (0.64–1.37)	0.721	1.06 (0.76–1.46)	0.740	0.611
SAT	1.06 (0.78–1.45)	0.709	0.99 (0.74–1.34)	0.966	0.758
VAT	0.77 (0.44–1.35)	0.363	1.04 (0.76–1.43)	0.810	0.353
SM	1.20 (0.64–2.25)	0.576	1.04 (0.68–1.58)	0.873	0.683
SMI	1.12 (0.58–2.16)	0.728	1.11 (0.77–1.60)	0.570	0.977
IMAT	0.99 (0.69–1.42)	0.958	1.04 (0.78–1.39)	0.781	0.818
**Overall Survival**					
	**Female**		**Male**		
	HR * 95% CI	***p***	HR * 95% CI	***p***	**Interaction *p***
BMI	0.72 (0.49–1.07)	0.101	0.99 (0.7–1.39)	0.957	0.224
SAT	0.88 (0.64–1.22)	0.457	0.98 (0.71–1.35)	0.894	0.657
VAT	0.71 (0.39–1.28)	0.253	0.93 (0.67–1.27)	0.634	0.425
SM	1.37 (0.69–2.71)	0.366	0.77 (0.49–1.21)	0.263	0.143
SMI	1.48 (0.74–2.96)	0.266	0.80 (0.54–1.20)	0.288	0.126
IMAT	0.70 (0.46–1.05)	0.086	1.30 (0.94–1.79)	0.108	0.013

Models adjusted for age, sex, treatment, ECOG status and LDH level. * HRs are for the 75th vs. the 25th percentiles of the distribution of each body composition measure. BMI: body mass index; CI: confidence interval; ECOG: Eastern Cooperative Oncology Group; HR: hazard ratio; IMAT: intermuscular adipose tissue; LDH: lactate dehydrogenase; SAT: subcutaneous adipose tissue; SM: skeletal muscle; SMI: skeletal muscle index; VAT: visceral adipose tissue.

## Data Availability

Restrictions apply to the availability of these data. Data were obtained from The Canadian Melanoma Research Network (CMRN). Further inquiries can be directed to the CMRN at info@melanomacanada.ca.

## Supplementary Materials

The following supporting information can be downloaded at: https://www.mdpi.com/article/10.3390/curroncol33070403/s1. Supplementary analyses in which we adjusted for cancer stage in addition to age, sex, treatment type, ECOG status and LDH level are provided in detail in Table S1: Overall hazard ratios for disease progression and death from survival models; Table S2: Body composition and bmi association with patient outcomes by treatment type for male and female patients combined; Table S3: Body composition and bmi association with patient outcomes by sex for both treatment groups combined; and Figure S1: Hazard functions for death in male and female patients as a function of IMAT, derived from an interaction model and combining both treatments. The hazard functions are expressed relative to the hazard for a male patient with IMAT at its 25th percentile (the dotted point).
