# Supplementary material for: Body Composition and Melanoma Outcomes in Patients on Immunotherapy or Targeted Therapy: An Analysis from Canadian Melanoma Research Network
Source: Curr Oncol. 2026 Jul 6;33(7):403. doi: 10.3390/curroncol33070403 (PMC13407559; doi:10.3390/curroncol33070403)
Supplement: Supplementary file 1 [file curroncol-33-00403-s001.zip › curroncol-4296035-supplementary.pdf]

Supplementary material: Models adjusted for cancer stage in addition to age, sex, treatment type, ECOG status and LDH level

Table S1. Overall Hazard Ratios For Progression And Death From Survival Models.

	Progression		Death	
**Variable**	**HR* (95% CI)**	***P* value**	**HR* (95% CI)**	***P* value**
BMI	1.05 (0.81 - 1.36)	0.712	0.93 (0.71 - 1.21)	0.580
SAT	1.05 (0.84 - 1.31)	0.683	0.97 (0.77 - 1.22)	0.770
VAT	1.05 (0.79 - 1.41)	0.723	0.99 (0.72 - 1.35)	0.937
SM	1.23 (0.83 - 1.82)	0.312	1.05 (0.69 - 1.60)	0.815
SMI	1.21 (0.86 - 1.71)	0.271	1.05 (0.72 - 1.54)	0.786
IMAT	1.02 (0.81 - 1.29)	0.854	1.00 (0.77 - 1.29)	0.982

*** HR are for the 75th vs 25th percentiles of each body composition's distribution BMI, Body mass index; CI: Confidence interval; ECOG: Eastern cooperative oncology group; HR: Hazard ratio; IMAT, Intermuscular adipose tissue; LDH: Lactate dehydrogenase; SAT, Subcutaneous adipose tissue; SM, Skeletal muscle; SMI, Skeletal muscle index; VAT, Visceral adipose tissue.**

Table S2. Body Composition And BMI Association With Patients' Outcomes by Treatment Type, For Male and Female Patients Combined

	Progression Free Survival						
	Immunotherapy			Targeted Therapy			
	**HR***	**95% CI**	***P***	**HR***	**95% CI**	***P***	**Interaction *P***
BMI	1.19	0.85 -1.66	0.302	0.89	0.61 -1.31	0.555	0.247
SAT	1.14	0.87 -1.50	0.354	0.93	0.67 -1.29	0.654	0.328
VAT	1.02	0.69 -1.50	0.915	1.09	0.73 -1.65	0.666	0.800
SM	1.44	0.93 -2.23	0.106	0.94	0.57 -1.56	0.818	0.104
SMI	1.48	0.99 -2.20	0.056	0.86	0.52 -1.42	0.550	0.063
IMAT	1.00	0.74 -1.34	0.985	1.06	0.76 -1.48	0.744	0.783
	**Overall Survival**						
	**Immunotherapy**			**Targeted Therapy**			
	**HR***	**95% CI**	***P***	**HR***	**95% CI**	***P***	**Interaction *P***
BMI	1.10	0.78 -1.55	0.577	0.73	0.49 -1.09	0.127	0.118
SAT	1.10	0.84 -1.44	0.498	0.76	0.53 -1.10	0.147	0.106
VAT	1.25	0.85 -1.84	0.262	0.72	0.46 -1.12	0.148	0.052
SM	1.22	0.78 -1.89	0.383	0.69	0.38 -1.25	0.220	0.054
SMI	1.15	0.76 -1.75	0.497	0.82	0.45 -1.51	0.529	0.307
IMAT	1.10	0.80 -1.52	0.542	0.87	0.59 -1.26	0.454	0.307

***HR are for the 75th vs 25th percentiles of each body composition's distribution**
BMI: Body mass index; CI: Confidence interval; ECOG: Eastern cooperative oncology group; HR: Hazard ratio; IMAT, Intermuscular adipose tissue; LDH: Lactate dehydrogenase; SAT, Subcutaneous adipose tissue; SM, Skeletal muscle; SMI, Skeletal muscle index; VAT, Visceral adipose tissue.

Table S3. Body Composition And BMI Association With Patients' Outcomes by Sex, For Both Treatment Groups Combined.

	**Progression Free Survival**				
	**Female**		**Male**		
	**HR* 95% CI**	***P***	**HR* 95% CI**	***P***	**Interaction *P***
BMI	0.93 (0.63 - 1.36)	0.698	1.16 (0.83 - 1.62)	0.394	0.376
SAT	1.06 (0.78 - 1.45)	0.710	1.04 (0.76 - 1.41)	0.825	0.911
VAT	0.83 (0.47 - 1.45)	0.505	1.14 (0.82 - 1.59)	0.432	0.316
SM	1.29 (0.67 - 2.48)	0.440	1.2 (0.77 - 1.87)	0.419	0.840
SMI	1.23 (0.63 - 2.38)	0.546	1.21 (0.83 - 1.77)	0.327	0.969
IMAT	0.96 (0.67 - 1.37)	0.816	1.07 (0.8 - 1.42)	0.668	0.633
	**Overall Survival**				
	**Female**		**Male**		
	**HR* 95% CI**	***P***	**HR* 95% CI**	***P***	**Interaction *P***
BMI	0.72 (0.49 - 1.07)	0.106	1.18 (0.81 - 1.71)	0.398	0.080
SAT	0.89 (0.64 - 1.23)	0.468	1.06 (0.76 - 1.49)	0.729	0.450
VAT	0.74 (0.41 - 1.35)	0.328	1.09 (0.76 - 1.56)	0.641	0.274
SM	1.48 (0.72 - 3.04)	0.291	0.92 (0.57 - 1.48)	0.736	0.250
SMI	1.76 (0.86 - 3.6)	0.120	0.9 (0.59 - 1.37)	0.634	0.100
IMAT	0.64 (0.43 - 0.96)	0.031	1.39 (1.01 - 1.92)	0.046	0.002

***HR are for the 75th vs 25th percentiles of each body composition's distribution**
BMI: Body mass index; CI: Confidence interval; ECOG: Eastern cooperative oncology group; HR: Hazard ratio; IMAT, Intermuscular adipose tissue; LDH: Lactate dehydrogenase; SAT, Subcutaneous adipose tissue; SM, Skeletal muscle; SMI, Skeletal muscle index; VAT, Visceral adipose tissue.

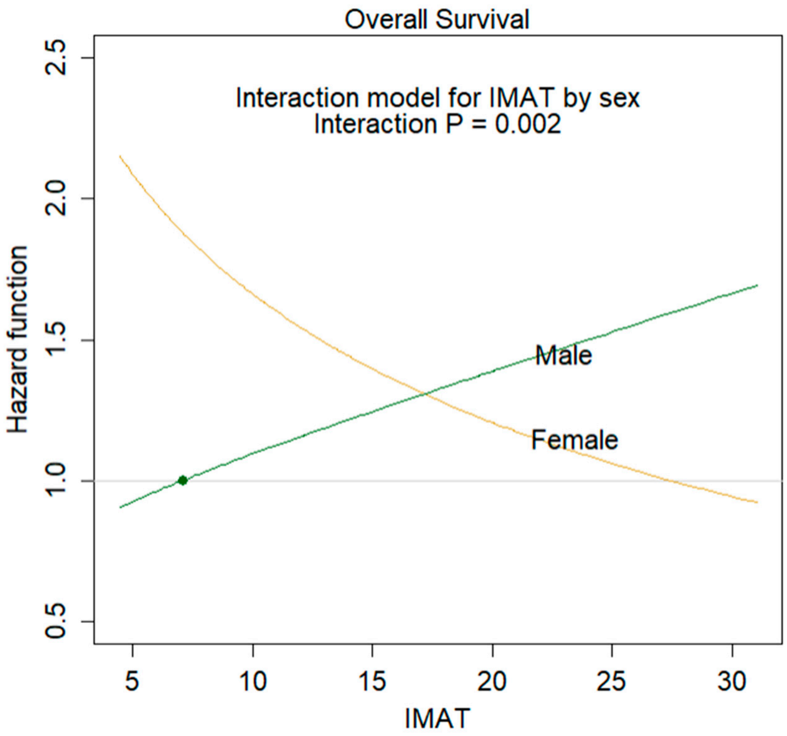

Figure S1. Hazard Functions For Death For Male And Female Patients as A Function of IMAT, Derived From An Interaction Model and Combining Both Treatments. The Hazard Functions Are Expressed Relative To The Hazard For A Male Patient With IMAT At Its 25th Percentile (The Dotted Point).
